# In silico analysis of molecular mimicry between human aquaporin 3, *Aspergillus fumigatus* aquaporin and aquaporins from allergic sources

**DOI:** 10.12688/f1000research.142843.1

**Published:** 2024-04-23

**Authors:** Andrés Sánchez, Yaquelin Padilla, Adriana Lorduy, Jorge Sanchez, Marlon Munera, Claudia Baena, Carlos Bernal, Juan Urrego

**Affiliations:** 1 Research Group of Pharmaceutical, Cosmetic, and Food Technology (GIFTCA), University of Cartagena, Cartagena, Bolívar, Colombia; 2 Clinical and Experimental Allergy Group (GACE), University of Antioquia, Medellín, Antioquia, Colombia; 3 Group of Research Medicine (GINUMED), Rafael Nunez University Corporation, Cartagena, Bolívar, Colombia

**Keywords:** Atopic dermatitis, in silico, molecular mimicry, Aspergillus fumigatus, aquaporins.

## Abstract

**Background:**

Atopic dermatitis (AD) is a chronic inflammatory skin condition that has a significant impact on quality of life. The immune response and allergy symptoms in AD are triggered by the recognition of specific allergens by IgE antibodies. Cross-reactivity can lead to auto-IgE responses, potentially worsening AD symptoms. Our research aimed to enhance our understanding of allergenic sources, including A. fumigatus, and their role in AD. We focused on molecular mimicry between human AQP3 and A. fumigatus aquaporin.

**Methods:**

In our in-silico analysis, we compared the amino acid sequences of human aquaporin 3 (AQP3) and A. fumigatus aquaporin with 25 aquaporins from various allergenic sources, sourced from the UniProt and NCBI databases. Phylogenetic relationship analysis and homology-based modeling were conducted. We identified conserved antigenic regions located within the 3D structures.

**Results:**

The global identity levels among the studied aquaporins averaged 32.6%. One antigenic site exhibited a remarkable local region, with a conserved identity of 71.4%. We categorized the aquaporins into five monophyletic clades (A–E), with group B showing the highest identity (95%), including six mammalian aquaporins, including AQP3. When comparing *A. fumigatus* aquaporins, the highest identity was observed with *Malassezia sympodialis* at 35%. Both human and A. fumigatus aquaporins have three linear and three discontinuous epitopes.

**Conclusions:**

We identified potential linear and conformational epitopes of AQP3, indicating a possible molecular mimicry between humans and *A. fumigatus* aquaporins. This suggests autoreactivity and potential cross-reactivity, although further validation using in vitro and in vivo experiments is required.

## Introduction

Atopic dermatitis (AD) is a chronic and recurrent inflammatory skin disorder characterized by symptoms, such as eczema, erythema, persistent itching, and continuous skin damage. Over the past 30 years, the prevalence of AD has shown a notable rise, affecting between 5% and 20% of the child population and approximately 7% of adults in industrialized countries. ^1^^,^^2^ The pathogenesis of AD is not completely clear; however, allergens can activate an intense Th2 response. ^3^ Allergenic sources can vary depending on the population; for example, house dust mites are the main source of sensitization in tropical countries, whereas in Europe, pollen is the main trigger for allergies. In addition, depending on the environmental conditions, it is possible to be exposed to different species; therefore, epidemiological studies have shown variations in the frequencies of sensitization. ^4^ The severity of the disease is influenced by several factors, including compromised skin barrier integrity due to reduced levels of filaggrins and ceramides as well as an elevated presence of water channels such as aquaporin, which drives the skin, making the patient more susceptible to atopic dermatitis. ^5^ In general, allergy management includes allergen avoidance, pharmacotherapy, and immunotherapy, where only the latter has a beneficial effect that lasts for years and allows for a reduction in anti-inflammatory treatment. ^6^^–^^8^

In addition to other environmental factors, the skin microbiome plays a pivotal role in AD development. *Aspergillus fumigatus* is an opportunistic pathogen in both poikilothermic and homeothermic animals. In humans, this pathogen has been associated with many pathologies such as invasive cutaneous aspergillosis, saprophytic lung colonization, and aspergillomas. In addition, *A. fumigatus* can induce IgE-mediated allergic diseases including rhinitis, allergic sinusitis, asthma, hypersensitivity pneumonitis, and AD. ^9^^,^^10^ It has been characterized as an important allergenic source, and some allergens, including thioredoxins and cyclophilins, share homology with human proteins and are implicated in the autoreactive response in AD.

Despite the identification of sensitization to extracts from allergenic sources, such as fungi, the recognition of specific IgE sensitization to allergens is necessary to carry out a better diagnosis and immunotherapy. ^11^ However, this represents a challenge, because most fungal allergens cross-react with other taxonomically distant species, making it difficult to prove the clinical relevance of IgE reactivity against these allergens. In addition, it is important to consider that the evolution of AD varies greatly among patients with this disease and can be triggered by different factors. For example, in *A. fumigatus* and AD, studies suggest that specific IgE-mediated activation is induced not by multiple allergens and not by a few major allergens, as is the case with asthma and rhinitis. ^12^^,^^13^ Another problem presented by the diagnosis of allergic diseases associated with *A. fumigatus* is the lack of standardized allergens for preparation of molecular component tests. Therefore, the discovery of new fungal allergens and the identification of the prevalence of these new components may provide an opportunity to learn more about the pathology of atopic dermatitis, improving the diagnosis and treatment of this disease.

In recent years, aquaporin has become a candidate protein for the study of skin and allergic diseases such as AD, given its role in epidermal hydration and the transport of various substances such as glycerol, salts, and exocrine fluids, favoring dry skin and allergen leakage. ^14^ They have also been associated with various atopic diseases, such as glaucoma, cancer, epilepsy, obesity, epidermal hyperplasia, and neuromyelitis optical, among others. In AD, an increase in the expression of aquaporin 3 has been observed, which may be related to water loss and the severity of the disease, in contrast to healthy individuals who maintain a balanced expression of this protein in the basal, horny, and spinous layers. ^5^ The consequences of aquaporin 3 (AQP3) overexpression extend beyond cutaneous water retention as it becomes a target for the exacerbated immune response in AD inflammation. This hypothesis suggests that it may be recognized by human autoantibodies, which, due to the persistence of self-protein exposure, contribute to increased inflammation and chronicity of AD.

Additionally, this protein is present in most organisms with a wide variety of isoforms and functions throughout the taxonomic framework. Recently, aquaporin has been identified in highly allergenic species, such as house dust mites. In a study carried out by Mao *et al.,* describing the *Dermatophagoides farinae* proteome, two-dimensional immunoblotting was performed, which showed binding with IgE antibodies in a band that has the same isoelectric point and molecular weight of the aquaporin family, suggesting its possible role as an allergen. The objective of this study was to perform an *in silico* analysis to investigate the potential molecular mimicry between human aquaporins and those from *A. fumigatus*, as well as various allergenic sources.

## Methods

For the selection of aquaporins, the *Aspergillus fumigatus* (KEY82230) and human aquaporin 3 (Q92482) sequences of interest were compared with existing allergenic protein sequences using Allermatch ( https://allermatch.org/), an allergen comparative server that allows allergen sequence alignment; therefore, those with high identity values were chosen. The sequence pool was obtained from the UniProt ( https://www.uniprot.org/) and National Center for Biotechnology Information (NCBI) databases. Given the diversity in amino acid origins and lengths across the molecules under examination, we employed coverage and identity values to gauge the extent of their relationships. The identity and coverage values between the molecules used in this study were determined using the EMBOSS Needle ( https://www.ebi.ac.uk/Tools/psa/emboss_needle/), specialized for performing paired alignments, and the web server from Praline ( http://www.ibi.vu.nl/), which allowed for paired and multiple alignments. When carrying out the alignment, it was considered that the sequences were different and came from different sources; therefore, the alignment parameters were set to utilize the BLOSUM62 matrix, which primarily assesses evolutionary divergence, along with adjustments to the conditional scoring matrix. ^15^^,^^16^

### Phylogenetic analysis

To construct the phylogenetic tree, we conducted a study using “Molecular Evolutionary Genetic Analysis” (MEGA) version 11 program. We employed the neighbor-joining (R) reconstruction method, supported by 100 bootstrap repetitions, to ensure the reliability and robustness of the analysis. Evolutionary distances were computed using the Poisson correction method, which uses a comparison matrix to identify sequence similarities. This matrix was created based on all amino acid sequences of the selected allergens, with all empty gaps removed (full deletions). Branch length summation (SBL) was presented as a measure of overall comparison and homologies, providing insights into the number of nodes, their positions, and the formation of the evolutionarily closest sequence “clusters. ^15^^,^^17^

### 3D models of proteins

Aquaporin models were acquired from the Protein Data Bank and AlphaFold ( *Homo sapiens, Felis catus, Equus caballus, Bos taurus, Mus musculus, Malassezia sympodialis, Saccharomyces cerevisiae, Penicillium chrysogenum, Escherichia coli*). For *A. fumigatus* and *Cannis lupus familiaris* aquaporin 3D structures were modeled by homology using the Swiss Model server ( https://swissmodel.expasy.org/), and these models were refined in ModRefiner. ( https://zhanggroup.org/ModRefiner/), an algorithm for the fine-grained refinement of protein structures at the atomic level. ^18^

### Epitope prediction

Models were used to locate possible epitope regions predicted by the Ellipro server ( http://tools.iedb.org/ellipro/), which was used to predict linear and discontinuous epitopes in the aquaporins of *A. fumigatus*, (AQP3), *Cannis lupus familiaris, Felis catus, Equus caballus, Bos taurus, Mus musculus, Malassezia sympodialis, Saccharomyces cerevisiae, Penicillium chrysogenum* and *Escherichia coli.* The potential epitopes were selected under the criteria of a score ≥ 7, as this value evaluates how exposed the residue is and the binding capacity of the epitope with the paratope. ^15^^,^^17^

### Identification of aquaporin domains

The characterization of the protein domains was carried out using the Interpro web server ( https://www.ebi.ac.uk/interpro/); with this bioinformatic tool we obtained those domains with functional activity present in the protein sequence of the aquaporins studied. Subsequently, a review of the tertiary structure was carried out using the PyMOL program, which functioned as a molecular viewer, allowing identification of the location of the protein domains in the tertiary structure of each one.

## Results

### Aquaporins sequence alignments

A total of 25 aquaporin sequences from different sources were selected and compared with the *A. fumigatus* aquaporin and human aquaporin ( Table 1), the identity percentages of the aquaporins from different sources Vs *A. fumigatus* aquaporin and AQP3 are shown in Table 2. Notably, human AQP3 exhibited maximum values of identity and similarity of 95.5% and 98.6%, respectively, with aquaporin allergy sources. When comparing *A. fumigatus* aquaporins and AQP3, an identity of 32.6% and a similarity of 47.5% were observed, indicating a significant degree of homology between them. Regarding the AQP of *A. fumigatus*, the highest identity values were observed in the AQP of *M. simpodialies* (35%), followed by the mammalian species that also had greater identity with human AQP3, such as *C. familiaris* (33, 9%), *Felis gatus* (33, 10%), *Mus musculus* (33, 10%), and Bos taurus (32, 80%). The lowest identity and similarity results were obtained with apple ( *Malus domestica*), with values of 0.2% and 0.9%, respectively.

**Table 1.** Aquaporins studies.

Organisms	Protein	Uniprot/NCBI Entry	Aminoácidos
*Aspergillus fumigatus*	aquaglyceroporin	KEY82230	335
*Homo sapiens*	Aquaporin-3	Q92482	292
*Dermatophagoides pteronyssinus*	Aquaporin	A0A2D1VL80	265
*Dermatophagoides farinae*	Aquaporin	A0A2C9PGE6	259
*Blomia tropicalis*	Aquaporin	A0A1Z1W2B1	256
*Euroglyphus maynei*	Aquaporin-like protein	A0A1Y3AUR4	289
*Malus domestica*	Aquaporin	H2EIG5	144
*Vitis vinífera*	Aquaporin TIP4-1	A0A438K8X4	196
*Actinidia deliciosa*	Aquaporin PIP2-1	A0A1X9ZIG1	283
*Citrus sinensis*	Putative aquaporin TIP13	A0A067DDA2	251
*Cannabis sativa*	Uncharacterized protein	A0A7J6FF47	250
*Blatella germanica*	Aquaporin TIP3-1	A0A2P8XIM1	200
*Periplaneta americana*	Putative glycerol diffusion channel	D0J8Y8	247
*Penaeus vannamei*	Aquaporin	A0A3R7PEU6	308
*Canis lupus familiaris*	Aquaporin-3	E2RI15	292
*Felis catus*	Aquaporin-3	A0A2I2UBV1	292
*Equus caballus*	Aquaporin-3	F6TFP8	419
*Bos Taurus*	Aquaporin-3	Q08DE6	292
*Mus musculus*	Aquaporin-3	Q8R2N1	292
*Alternaria alternata*	Aquaporin	A0A177DNX7	408
*Malassezia Sympodialis*	Putative channel-like protein	A0A1M7ZZV8	291
*Saccharomyces cerevisiae*	Aquaglycerol porin AQY3	P43549	646
*Penicillium chrysogenum*	Aquaporin-3	A0A167PPH9	279
*Penicillium rubens*	Pc12g14590 protein	B6H130	306
*Escherichia coli*	Glycerol uptake facilitator protein	P0AER0	281
*Ascaris lumbricoides*	Aquaporin	A0A0M3I310	267
*Staphylococcus aureus*	Glycerol uptake facilitator protein	AYU99483	247

**Table 2.** Identity and similarity between AQP3, *A. fumigatus* aquaporin and aquaporins from allergen sources.

Sources	AQP3			*Aspergillus Fumigatus*		
	Identity (%)	Similarity (%)	Score	Identity (%)	Similarity (%)	Score
*Aspergillus fumigatus*	32,6	47,5	492,5	-	-	-
*Dermatophagoides pteronyssinus*	18,1	32,2	42,0	15,50	25,5	41
*Dermatophagoides farinae*	31,5	48,7	386,0	22,90	36	291
*Blomia tropicalis*	19,6	32,7	44,5	13,70	20	29
*Euroglyphus maynei*	29	46	360	21,80	34,6	252,5
*Malus domestica*	1,7	3,2	14,5	0,20	0,9	3
*Vitis vinifera*	1,7	30,5	175	18,20	27,7	149,5
*Actinidia deliciosa*	21,2	32,9	218,5	24,30	38	247
*Citrus sinensis*	25,4	38,4	193,5	18,80	29,6	167
*Cannabis sativa*	24,6	39	190,5	19,50	34,7	167
*Blatella germanica*	22,6	33,4	141,0	15,90	23,2	84,5
*Periplaneta americana*	27,1	44,4	332	24,20	34	330
*Penaeus vannamei*	14,9	28,5	80,5	13,00	20,2	27
*Canis lupus familiaris*	72,9	75,9	1294	33,90	50,3	468,5
*Felis catus*	95,5	97,6	1484	33,10	48,4	508,5
*Equus caballus*	66,6	68,7	1497	27,90	42,7	498
*Bos taurus*	94,9	98,3	1486	32,80	48,1	497,5
*Mus musculus*	95,5	98,6	1493	33,10	48,1	506,5
*Alternaria alternata*	15,6	25,4	214	14,50	22,6	205
*Malassezia Simpodiales*	33,1	49,5	401,5	35,90	49,6	516
*Saccharomyces cerevisiae*	15,8	25,1	516	22,60	31,4	668,5
*Penicillium chrysogenum*	33,2	50,9	377,5	3,50	43,4	477
*Penicillium rubens*	24,6	35,2	205	20,90	32,8	144,5
*Escherichia coli*	35,6	53,2	475,5	31,70	45,2	436,5
*Ascaris lumbricoides*	9,9	16,1	30,5	10,40	16,7	17,5
*Staphylococcus aureus*	31,7	43,6	377,5	27,70	37,4	337,5

### Phylogenetic analysis

**Figure 1.**
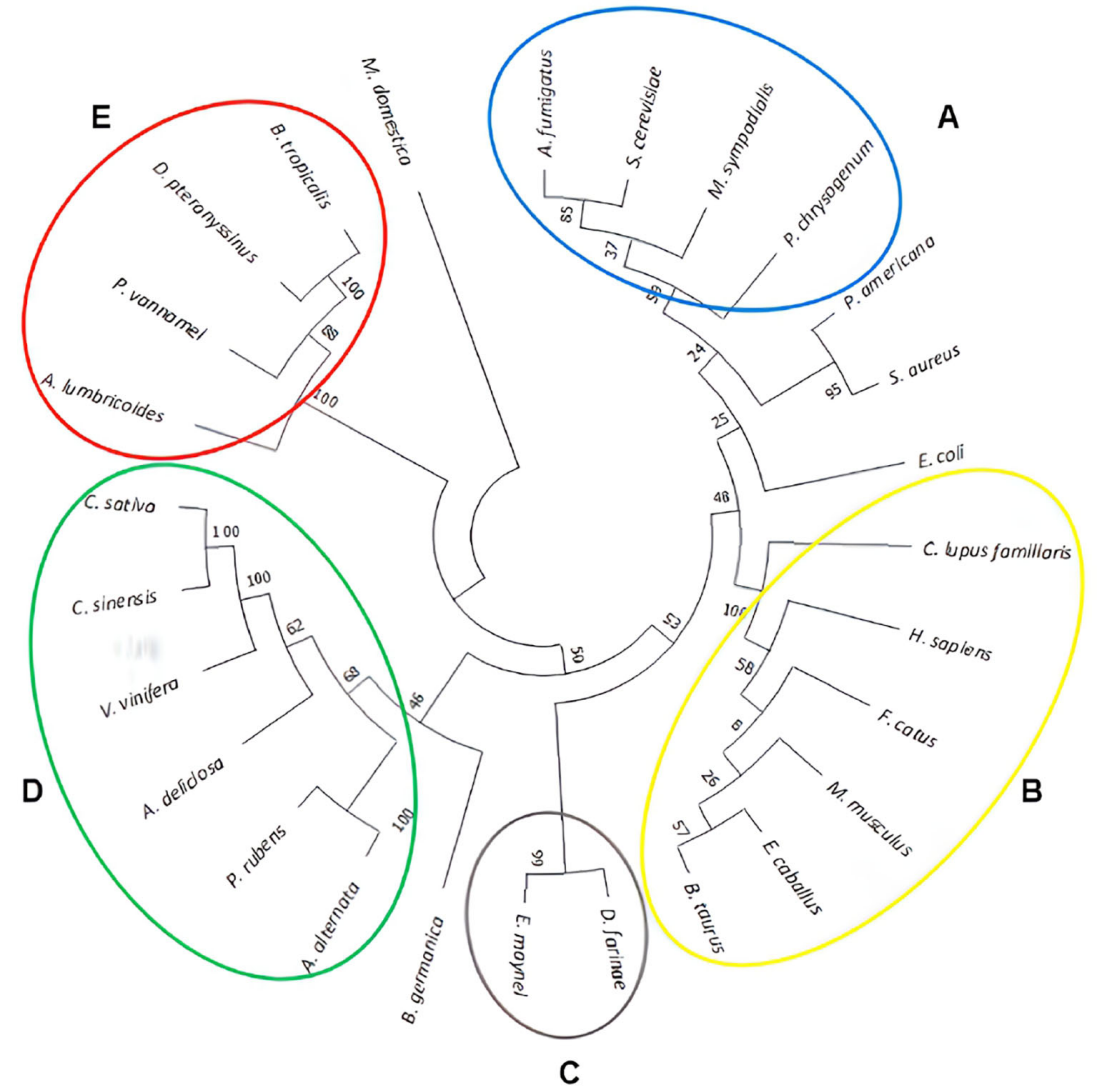
Phylogenetic tree based on amino acid sequences of the studied aquaporins. The sums of branch lengths for an optimal tree totaled 12.5. The tree reveals the formation of five groups, designated as letters from A to E.

Phylogenetic relationships were determined using the neighbor-joining method, incorporating all 27 amino acid sequences. The resulting phylogenetic tree is presented in a circular format in Figure 1. Evolutionary distances were calculated using the Poisson correction method and were exprex|ssed as the number of amino acid substitutions per site. The tree visually represents the grouping and arrangement of the analyzed aquaporins, facilitating a comprehensive comparison and revealing their interrelatedness.

Based on branch distances, the phylogenetic tree presented the formation of five distinct groups, denoted as letters A to E. Multiple sequence alignments were performed using the Praline server to further explore the similarities within each group. Group A consisted of fungal species with a shared identity of 39%, whereas group B comprised mammalian aquaporins with a high identity of 95%. Group C was composed of two mite species with an identity of 53%, group D encompassed plant sources with 40% identity, and group E included pathogens and mites with an identity of 31%. Notably, this analysis revealed significant allergen sources in tropical regions, such as *D. pteronissinus* and *B. tropicalis.*

Species such as *P. americana, S. aureus, E. coli, B. germanica,* and *M. domestica* did not fit within any specific group because of their substantial branch distances from the root of clustering. Consequently, they were categorized into independent groups. Among the identified groups, those most closely related to the aquaporins of *A. fumigatus* and humans were found in groups A and B. Additionally, noteworthy proximity was observed between *E. coli* and *D. farinae* in group B.

**Figure 2.**
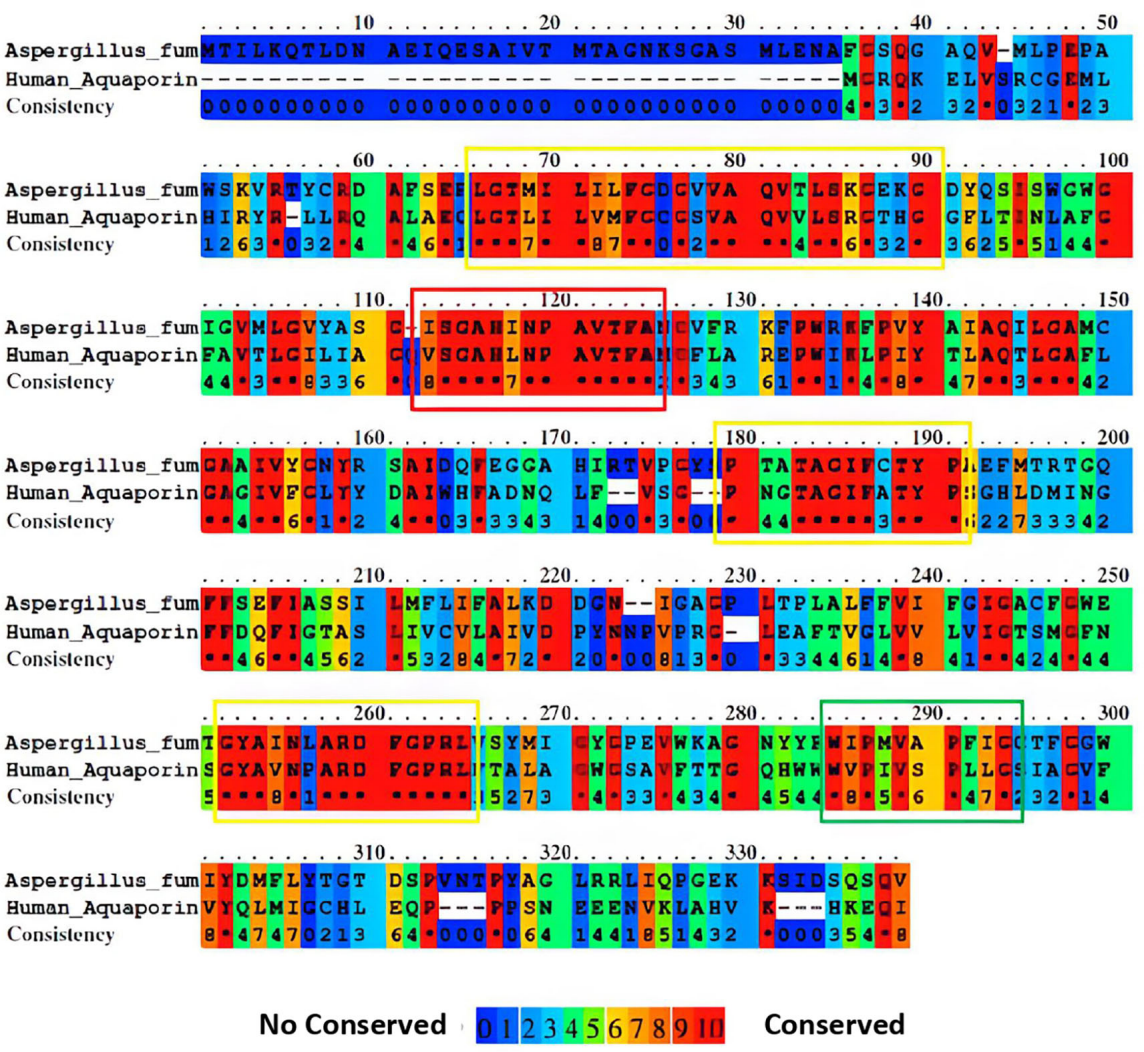
Sequence alignment of aquaporins from *A. fumigatus* and humans was performed using Praline. The colors used in the alignment represent the level of sequence conservation, with blue indicating lower conservation and red indicating higher conservation. In the accompanying image, highlighted boxes denote the most conserved regions in the sequence, with each color (red, yellow, and green) representing the degree of conservation in descending order.

The guidance provided by the phylogenetic tree and the results of paired alignments of the EMBOSS needle and aquaporin sequences were selected with a score ≥380. This threshold ensured an identity greater than 30% for each aquaporin sequence. Subsequently, paired alignments of the selected aquaporins against *A. fumigatus* were performed using Praline. This approach provides a more visually informative result, enabling a better understanding of the conserved regions in each sequence ( Figure 2). By employing this screening process, we can identify more conserved sequences, thus increasing the likelihood of identifying regions that may contain potential epitopes.

### Aquaporin 3D models

**Figure 3.**
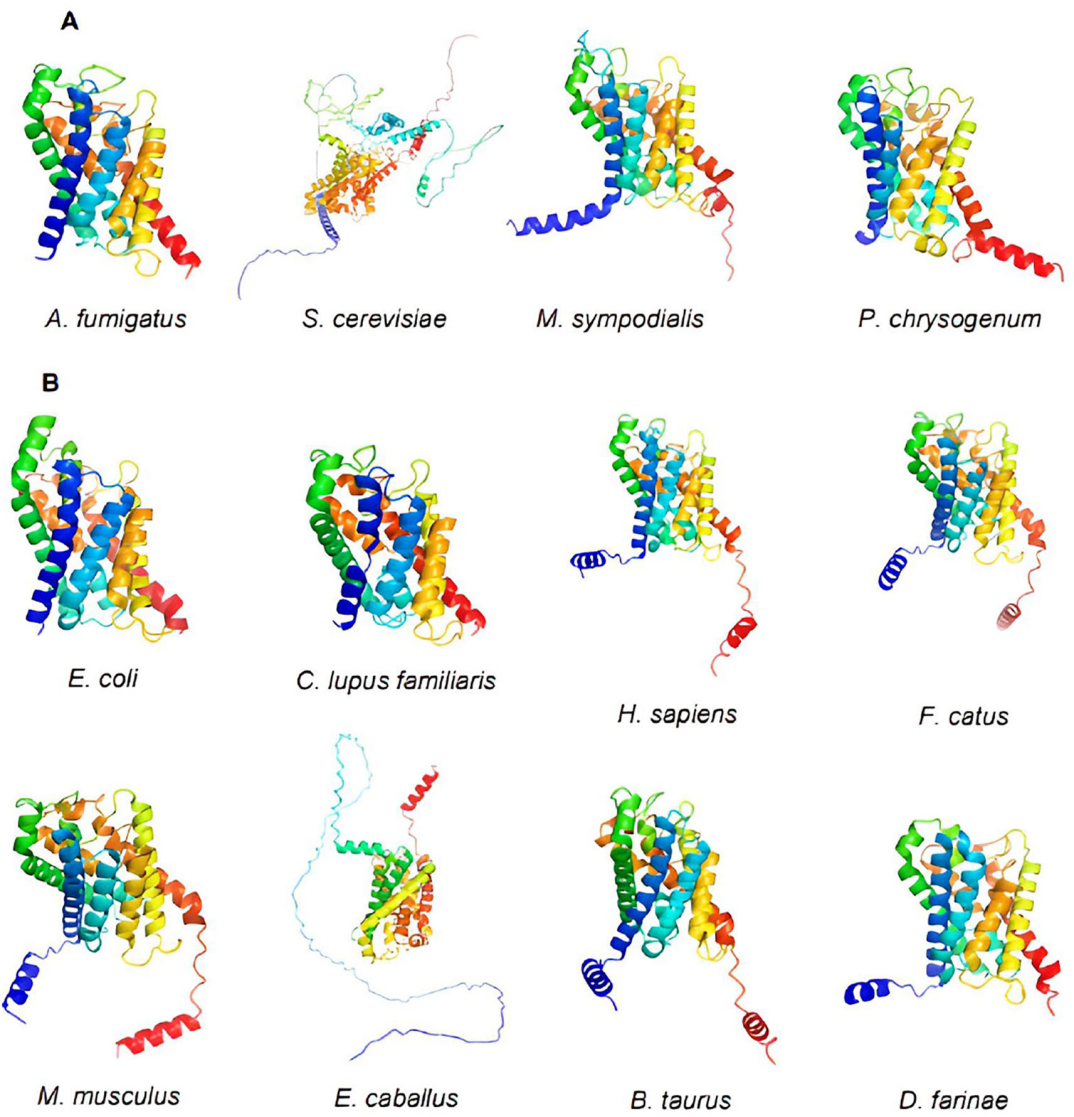
Structures of aquaporins obtained from sources including PDB, Uniprot, AlphaFold, and Swiss-model. Their clustering corresponds to the arrangement in the phylogenetic tree. (A) This cluster comprises aquaporins from fungi. (B) Aquaporins from animals, except for *E. coli*, belong to this group.

Using PyMOL, we visualized the structures of the 12 selected aquaporin proteins. They were represented in the form of “cartoons” and were color-coded using the spectrum. These models exhibit the characteristic structure of aquaporins, consisting of six alpha helices connected by extracellular and intracellular “loops” that form the water and substance transport channel ( Figure 3).

Moreover, certain structures display distinctive extensions in their amino acid chains, resulting in variations in their tertiary structures. Examples include *S. cerevisiae* and *E. caballus.* Consequently, during epitope exploration, most of these regions were exposed, leading to the exclusion of these sequences as potential allergenic sources due to the absence of epitopes in the conserved regions of interest. This minimized the likelihood of cross-reactivity.

### Prediction of linear and discontinuous epitopes

**Figure 4.**
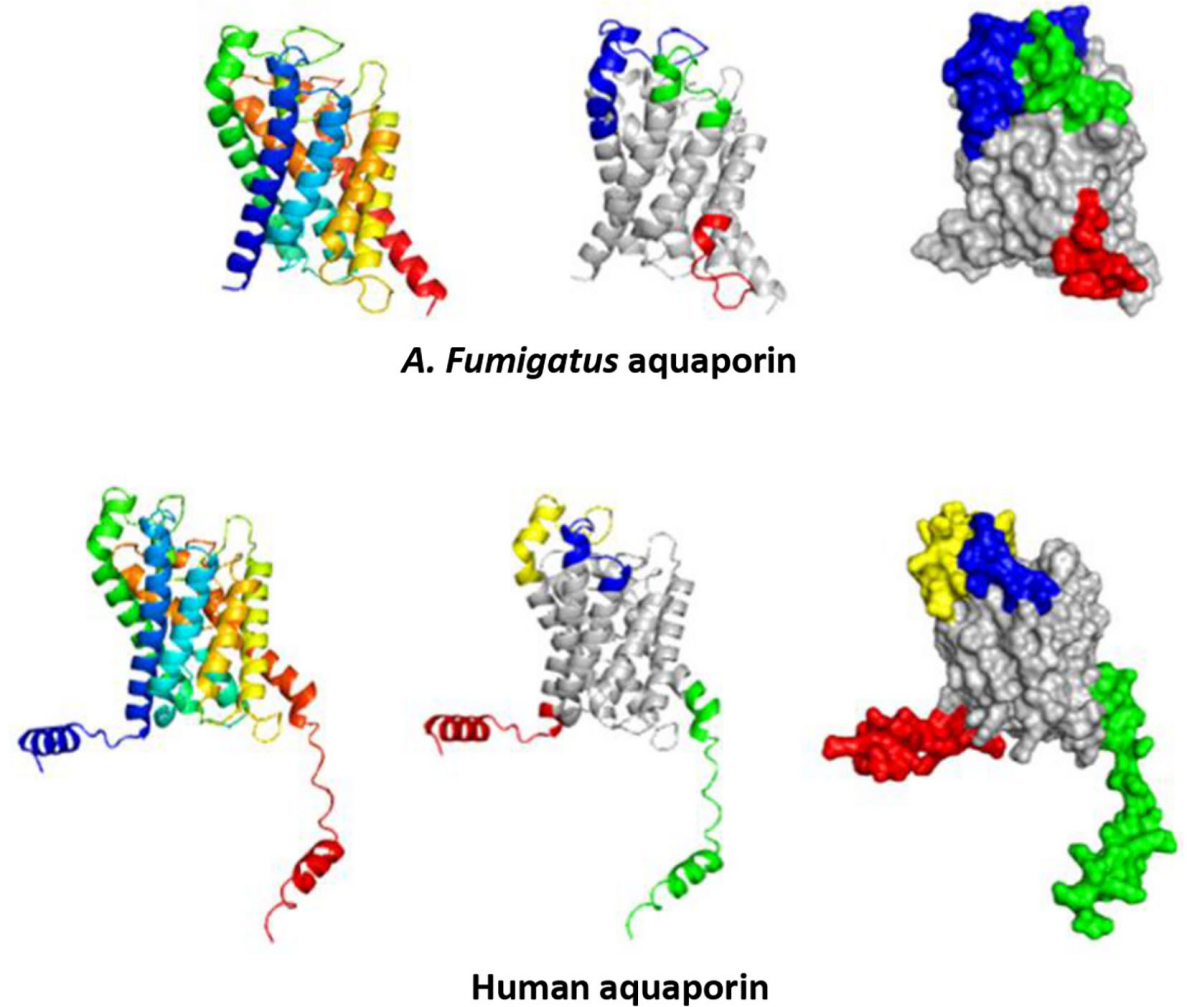
Depiction of predicted linear epitopes. All epitopes are color-coded on the surface models. Red, green, blue, and yellow correspond to the highest values, respectively.

**Figure 5.**
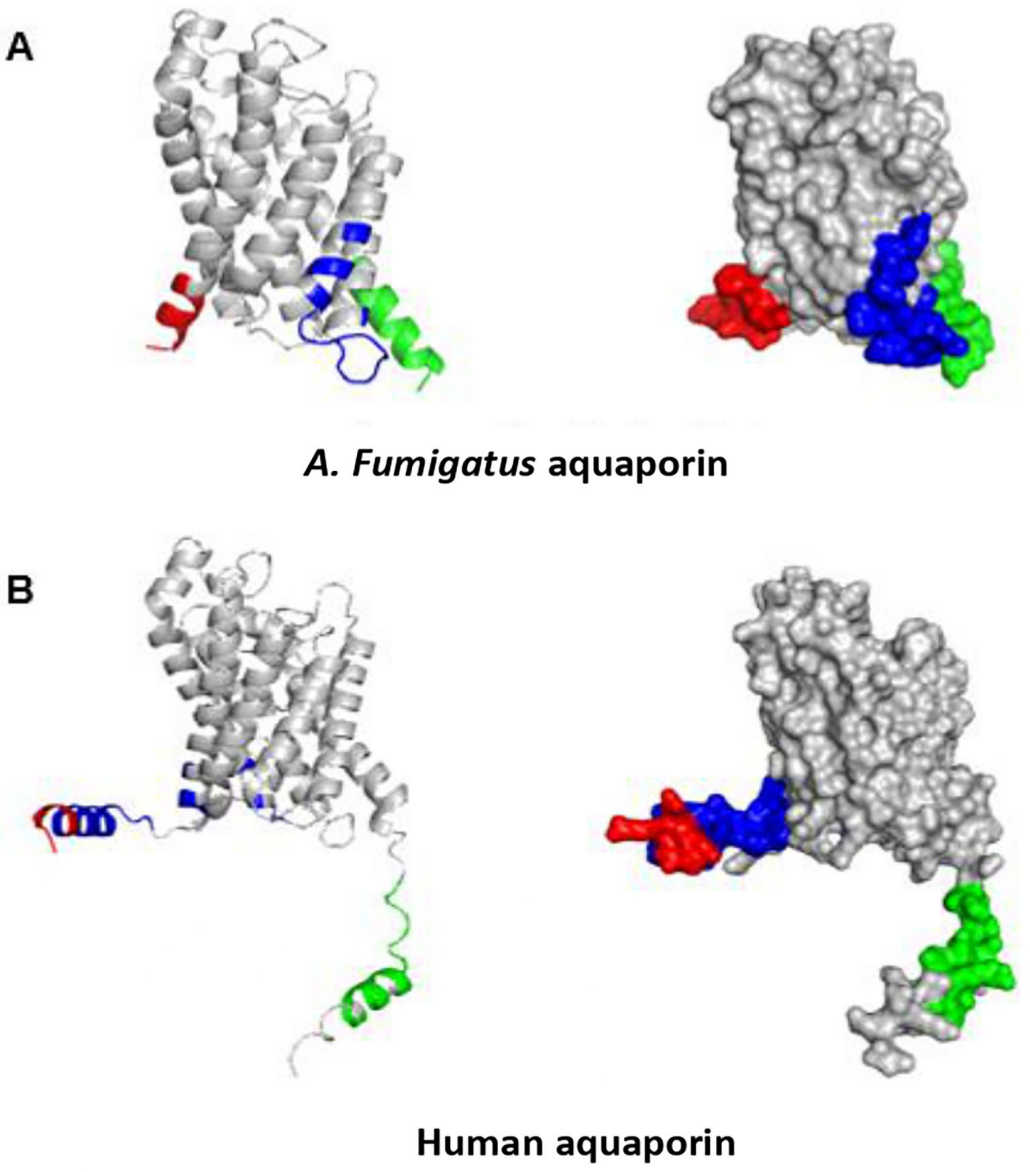
Representation of predicted discontinuous epitopes from *A. fumigatus* aquaporin and AQP3. All epitopes are highlighted with color on the surface models, where red, green, and blue signify the highest values, respectively.

Using the 3D structures of *A. fumigatus*, *Homo sapiens, Dermatophagoides farinae, Canis lupus familiaris, Felis catus, Equus caballus, Bos taurus, Mus musculus, Malassezia Sympodialis, Saccharomyces cerevisiae, Penicillium chrysogenum, and Escherichia coli*, we predicted the linear and discontinuous epitopes for each aquaporin ( Table 3). We utilized PyMOL to analyze the identified linear and conformational epitopes, and thoroughly examined the distinct regions within each of these 3D structures. These epitopes are marked with the representative colors in the 3D structure. Although this characterization was performed for all 12 studied aquaporins, this work focused only on the epitopes found in the aquaporins of interest ( *A. fumigatus* and AQP3), as shown in Figures 4 and 5.

**Table 3.** Lineal and discontinuous epitotpes from *A. fumigatus* and human aquaporin 3 (AQP3).

*Aspergillus Fumigatus* aquaporin						
Lineal Epitopes						
No.	Chain	Start	Final	Peptide	N. Residues	Score
1	A	218	231	DDGNIGAGPLTPLA	14	0,763
2	A	80	93	QVTLSKGEKGDYQS	13	0,7
3	A	151	180	AIVYGNYRSAIDQFEGGAHIRTVPGYSPTA	30	0,7
**Discontinuous Epitopes**						
1	A	54	61	A:R54, A:T55, A:Y56, A:C57, A:R58, A:D59, A:A60, A:F61	8	0,869
2	A	211	306	A:F211, A:F293, A:G295, A:W296, A:I297, A:Y298, A:D299, A:M300, A:F301, A:L302, A:Y303, A:T304, A:G305, A:T306	14	0,815
3	A	215	234	A:A215, A:D218, A:D219, A:G220, A:N221, A:I222, A:G223, A:A224, A:G225, A:P226, A:L227, A:T228, A:L230, A:A231, A:F234	15	0,763
**Human Aquaporin 3**						
**Lineal Epitopes**						
1	A	1	23	MGRQKELVSRCGEMLHIRYRLLR	23	0,872
2	A	259	292	FVYQLMIGCHLEQPPPSNEEENVKLAHVKHKEQI	34	0,856
3	A	45	58	QVVLSRGTHGGFLT	14	0,709
4	A	120	143	FGLYYDAIWHFADNQLFVSGPNGT	24	0,702
**Discontinuous Epitopes**						
1	A	1	5	A:M1, A:G2, A:R3, A:Q4, A:K5	5	0,957
2	A	271	285	A:Q271, A:P272, A:P273, A:P274, A:S275, A:N276, A:E278, A:E279, A:N280, A:V281, A:K282, A:L283, A:A284, A:H285	14	0,914
3	A	6	103	A:E6, A:L7, A:V8, A:S9, A:R10, A:C11, A:G12, A:E13, A:M14, A:L15, A:H16, A:I17, A:L21, A:L22, A:R23, A:L26, A:E96, A:P97, A:I99, A:P102, A:I103	21	0,775

**Figure 6.**
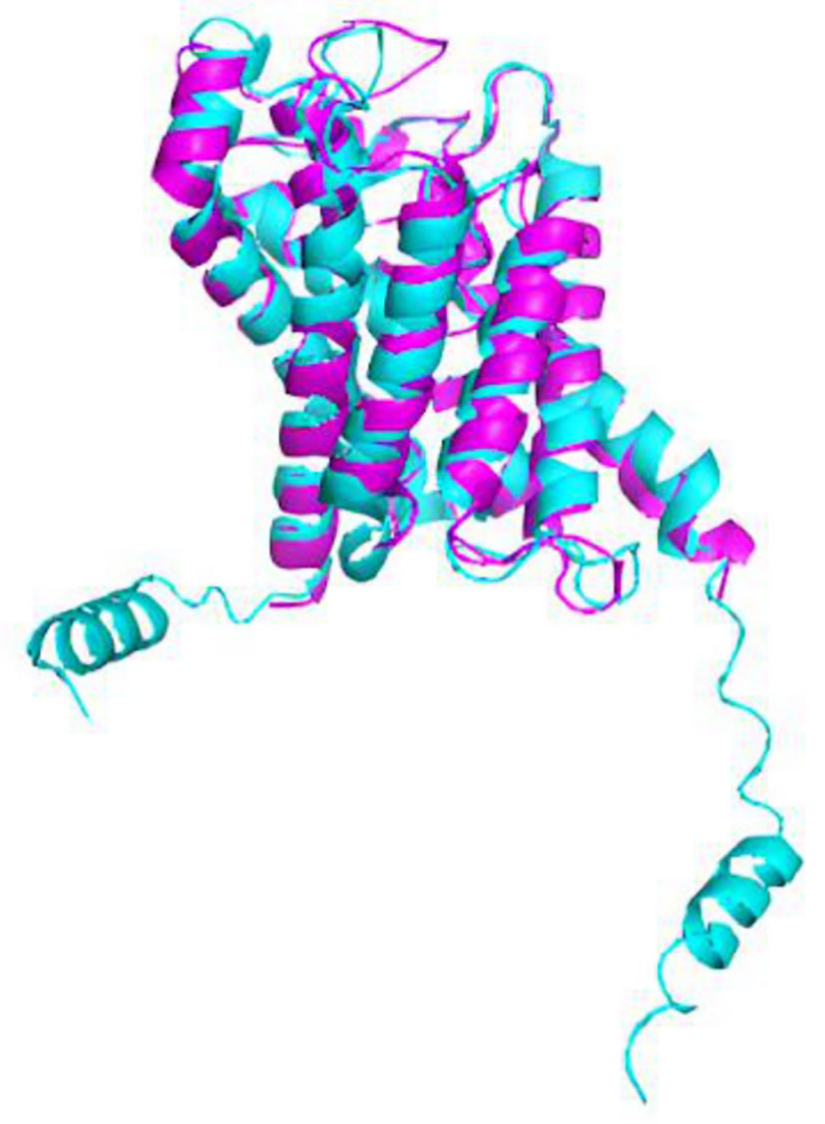
The structural overlap of *A. fumigatus* aquaporin (magenta) and human aquaporin AQP3 (cyan) is depicted in a cartoon model. The root-mean-square deviation (RMSD) is 1.003.

Furthermore, the PyMOL visualization program allowed us to calculate the root mean standard deviation (RMSD), which measures the structural similarity between two aligned objects, specifically the proteins of interest ( Figure 6). The closer the RMSD value is to zero, the stronger the molecular similarity between the proteins. We obtained an RMSD value of 1.003, indicating a significant molecular similarity.

**Figure 7.**
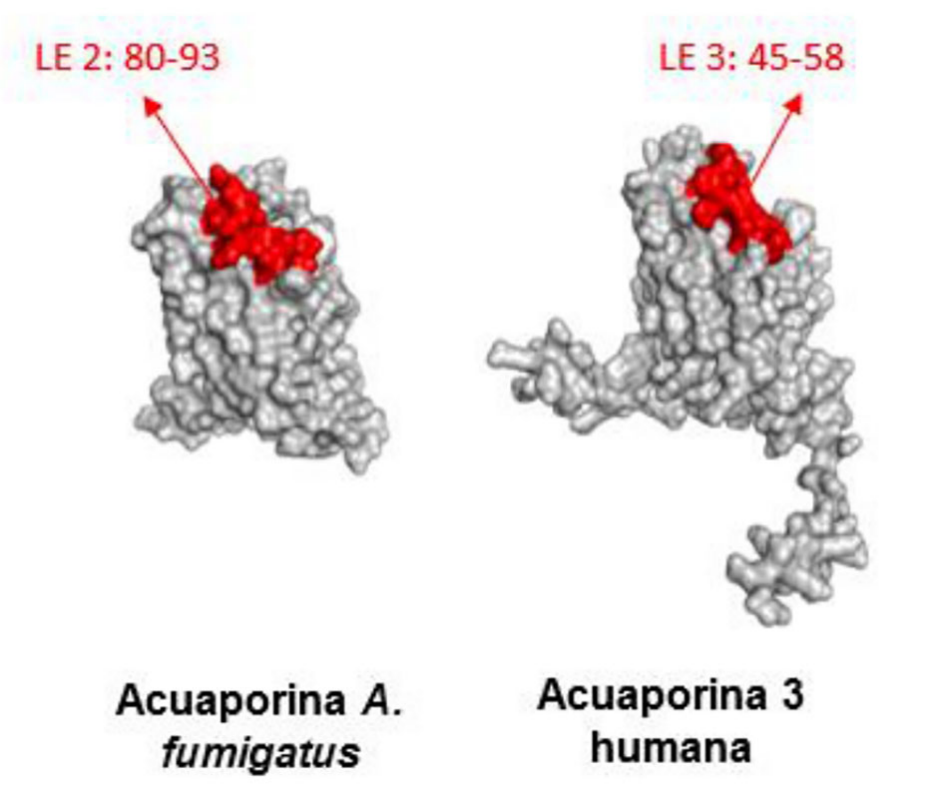
Potential similar antigenic patches in linear epitopes of *A. fumigatus* aquaporin and AQP3.

**Figure 8.**
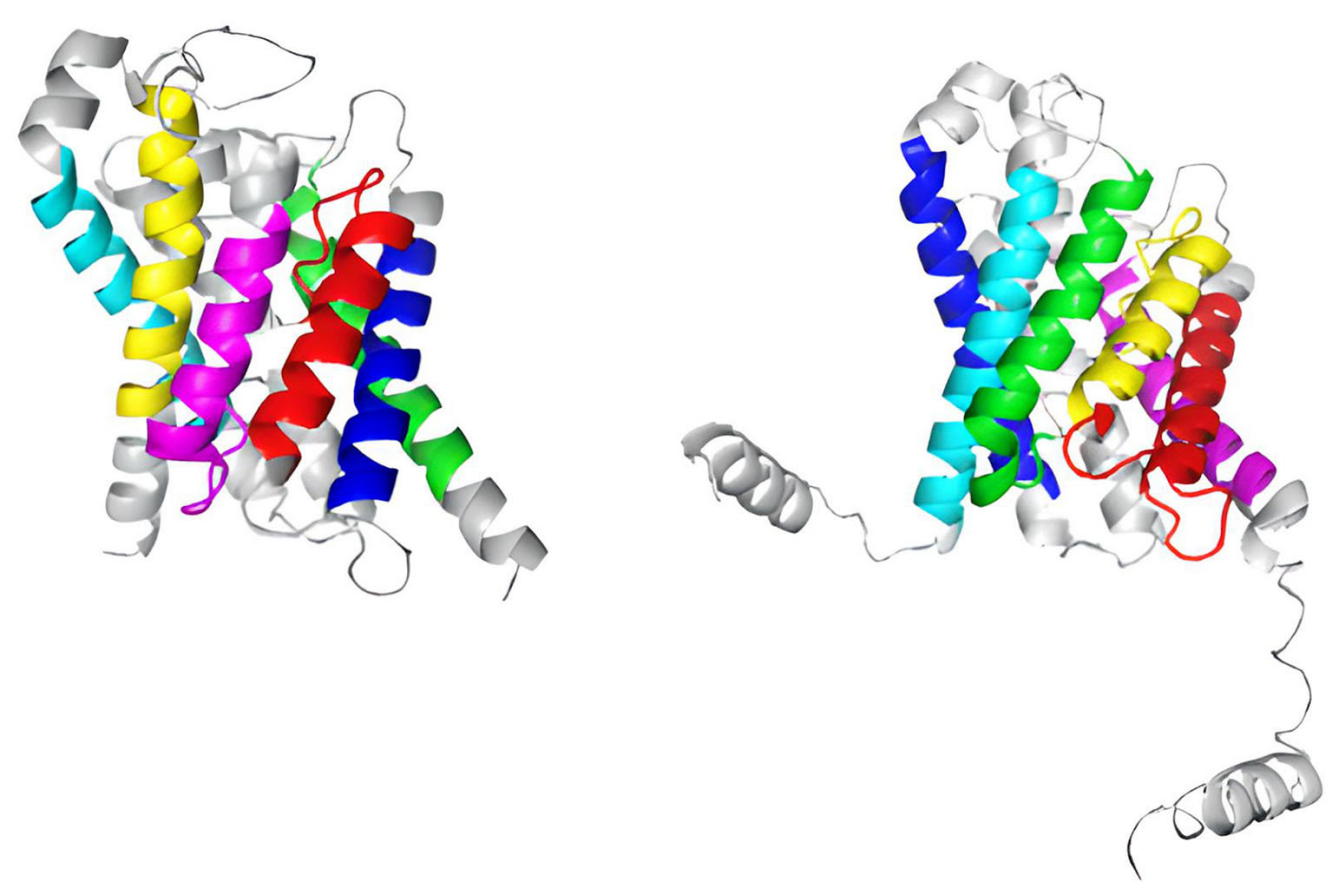
Identification of transmembrane functional activity domains in the aquaporin proteins of human AQP3 and the fungus *A. fumigatus* through color-coded assignment in their secondary structure.

In addition, we identified epitopes that contained conserved sequences. In the case of fungal aquaporins, Linear Epitope (LE) 2 was identified, whereas in human aquaporins, LE 3 was found. Individual analysis revealed highly conserved fragments with more than four residues. For fungal aquaporins, the conserved residue is 80QVTLSKG86, whereas for human aquaporins, it is 45QVVLSRG52. The percentage of identity and similarity between these fragments was 71.4% and 85.7%, respectively, suggesting that these residues were highly likely to be antigenic patches ( Figures 7 and 8).

## Discussion

Numerous diseases, including atopic dermatitis, bullous pemphigoid, chronic spontaneous urticaria, and multiple sclerosis, are associated with the presence of IgE autoantibodies. In the context of allergic responses intertwined with autoimmunity, researchers have categorized autoantigens that bind to IgE into three functional groups: 1) autoantigens that share sequence homology with environmental allergens, 2) autoantigens that lack sequence homology with known environmental allergens, and 3) chemically modified autoantigens. However, the identification of environmental allergens with homology to human proteins and their influence on the allergic response vary among these autoantigens, and their clinical significance often remains unclear. ^19^

Aquaporin is a promising protein candidate for studying cutaneous, autoimmune, and allergic diseases, including atopic dermatitis (AD), because of its involvement in epidermal hydration and transportation of various substances such as glycerol, salts, and exocrine fluids. These functions contribute to skin dryness, which, in turn, facilitates the filtration of allergens. ^14^ This protein is widely present in numerous organisms, and exhibits a diverse range of isoforms and functions across different taxonomic groups.

Recent research has identified the presence of aquaporins in allergenic species such as dust mites. ^20^ Mao et al. characterized the proteome of *Dermatophagoides farinae*, and two-dimensional immunoblotting revealed the binding of IgE antibodies to a band with the same isoelectric point and molecular weight as the aquaporin family, suggesting its potential role as an allergen. ^21^

On the other hand, in a systematic review, a total of 32 articles were examined to assess the role of the microbiota in atopic dermatitis. The microbiome profile revealed substantial variation in bacterial diversity along with an increased presence of fungi. Notably, there was diversification and expansion of species belonging to the genus *Aspergillus* within the fungal group, whereas a reduction in the number of *Malassezia* spp. was observed. Research has also demonstrated that *A. fumigatus* is a prominent source of sensitization in patients with severe atopic dermatitis. Additionally, the presence of new allergens from this species is believed to have a significant impact on the severity of the condition. ^22^

The comprehensive analysis of aquaporins provides guidance for considering other potential allergenic sources beyond the main aquaporins studied, namely AQP3 and *A. fumigatus* aquaporin. We conducted a comparative analysis of 25 aquaporin sequences from various sources, including *A. fumigatus* aquaporin and human aquaporin 3 (AQP3). A comparison between *A. fumigatus* aquaporin and AQP3 revealed an identity of 32.6% and similarity of 47.5%, indicating a relevant level of homology between these two proteins despite the evolutionary gap and taxonomic diversification among species. Notably, human AQP3 exhibited the maximum identity and similarity values of 95.5% and 98.6%, respectively. These high values could be attributed to the inclusion of aquaporins from mammalian allergen sources. Given that these species belong to a taxonomic group closer to humans, it is understandable that the APQs are more conserved.

Five clades were constructed for the phylogenetic analysis. A and B showed the highest identity with *A. fumigatus* aquaporins and AQP3. The alignment results suggest that divergence among aquaporins primarily occurs in smaller species, while it remains conserved in mammals. In addition, the presence of aquaporin isoforms, each with special functions and locations, such as AQP3, indicates functional and phenotypic variability among different cell types within the same organism. However, despite this diversity, the identity of aquaporins within the clades revealed values higher than 30%, particularly in clade B (95%), indicating that if the protein is recognized by antibodies, potential cross-reactivity among these species can occur.

Only a limited number of studies have investigated the relationship between aquaporins derived from allergenic sources and their potential relevance in human diseases. Zhou *et al.* conducted an assessment using transcriptomics to identify potential AQPs in *Blomia tropicalis* using molecular cloning techniques, followed by a comparative analysis of identity among mite aquaporin sequences and human aquaporins. The author identified five putative AQP-coding sequences, known as BlotAQP1-5, which were indexed into all three subgroups: AQPs, aquaglyceroporins, and superAQPs. BlotAQP1, BlotAQP2, and BlotAQP5 were clustered with the aquaglyceroporins hAQP3, hAQP7, hAQP9, and hAQP10, with a common sequence consensus ‘N-G-NPSRD-PRL.’ The molecular weight and isoelectric point of these molecules were consistent with the findings reported by Mao *et al.* in their two-dimensional electrophoresis study. ^23^ This observation suggests the potential recognition of aquaporins, specifically aquaglycerins such as AQP3, by IgE in patients with allergies. These results are consistent with those presented in our findings, where despite the evolutionary distance between species, there is an identity exceeding 30% and consensus sequences that may serve as potential antigenic patches.

In contrast, in regions where higher conservation was observed among the sequences, neither linear nor conformational epitopes were identified. This is attributed to the fact that these sequences are in the transmembrane region of the protein, which limits their accessibility to antibodies. However, despite the absence of epitopes, these conserved regions are interesting. During cell lysis caused by mechanical injury or microbial effects, these proteins can be linearly exposed, leading to the emergence of neoepitopes that can be recognized by IgE or phagocytic cells. ^24^

Although little is known about the role of AQP3 as an allergen or autoantigen, other aquaporins have been studied for their relevance as antigens in autoimmune diseases. Sagan *et al.* investigated the relevance of AQP4 as an autoantigen in neuromyelitis optica, recognized by T lymphocytes, in an animal model. ^25^ This aquaporin is a water channel expressed in astrocytes in areas in contact with the blood-brain barrier, and 75% of patients with neuromyelitis have antibodies capable of recognizing AQP4. Therefore, the relevance of human aquaporins found in other tissues, such as AQP3 in the skin, can be considered as potential autoantigens and, through molecular mimicry, triggers relevant skin diseases such as atopic dermatitis. ^26^ Evidence indicates cross-reactivity with aquaporins expressed by bacteria and mycobacteria, including *Escherichia coli* and Mycobacterium species, in relation to this specific autoantigen. ^27^ This indicated that antibodies targeting aquaporins from *A. fumigatus* could potentially recognize and target this autoantigen. In a previous study, we found that AQP4 exhibits only one predicted cross-reactive epitope. It has been postulated that the recognition of a single epitope by autoantibodies could trigger an epitope spreading mechanism, leading to the involvement of additional autoantigens in the immune response. ^28^ For both *A. fumigatus* aquaporins and AQP3, a total of four linear epitopes and three discontinuous epitopes were described. Structural superimposition highlighted a notable degree of similarity between the proteins, indicating that the epitopes could reside within the corresponding antigenic regions. This observation supports the idea of a potential molecular mimicry between these proteins.

It is important to acknowledge that our study has several limitations. In silico modeling and epitope prediction analyses are not definitive, and there may be variations in the actual structure compared with our proposed models. Nonetheless, bioinformatic analyses offer several advantages in directing research resources efficiently. They play a crucial role in the preliminary assessment of hypotheses and help to determine whether it is justified to proceed with in vitro or ex vivo experiments.

## Conclusion

During alignment of aquaporin sequences from A. fumigatus and AQP3, specific conserved epitopes were observed. Analysis revealed that the local sequence identity exceeded 70%, indicating molecular mimicry in these regions of both aquaporins. Further investigation of epitopes and domain identification revealed the most conserved region in transmembrane domains 12 and 14 of the respective aquaporins. This study represents a groundbreaking contribution to the exploration and analysis of aquaporin as a potential allergen and autoallergen in atopic dermatitis, as it is the first known study to focus on this subject. To confirm cross-reactivity, future in vitro studies are needed to demonstrate the IgE-binding capacity in these regions.

By acquiring knowledge regarding the sequences and structures of potential epitopes from different allergenic sources, we can modify and synthesize these molecules for future in vitro and in vivo studies. This not only expands the literature in the field of immunotherapy but also facilitates the diagnosis and treatment of autoimmune diseases such as atopic dermatitis. It is important to note that in silico tests optimize these studies by providing rapid results and circumventing the high costs associated with laboratory testing.

## Data Availability

Allermatch ( https://allermatch.org/) UniProt ( https://www.uniprot.org/) National Center for Biotechnology Information (NCBI) databases ( https://www.ncbi.nlm.nih.gov/). There is no further data associated with this article.
