# Supplementary material for: In silico analysis of molecular mimicry between human aquaporin 3, Aspergillus fumigatus aquaporin and aquaporins from allergic sources
Source: F1000Res. 2024 Sep 12;13:358. Originally published 2024 Apr 23. [Version 2] doi: 10.12688/f1000research.142843.2 (PMC11415755; doi:10.12688/f1000research.142843.2)
Supplement: Peer review history

# Reviewer report: Reviewer response for version 2

**DOI:** 10.5256/f1000research.171499.r323088

**Published:** 2024-09-20

**Authors:** Celso Eduardo Olivier

**Affiliations:** 1 American Institute Alergoimuno, Americana, Brazil

The version 2 is ready to be indexed. Congratulations!

Is the work clearly and accurately presented and does it cite the current literature?

Partly

If applicable, is the statistical analysis and its interpretation appropriate?

Not applicable

Are all the source data underlying the results available to ensure full reproducibility?

Yes

Is the study design appropriate and is the work technically sound?

Yes

Are the conclusions drawn adequately supported by the results?

Partly

Are sufficient details of methods and analysis provided to allow replication by others?

Yes

Reviewer Expertise:

Allergy and Immunology; non-IgE-mediated hypersensitivity

I confirm that I have read this submission and believe that I have an appropriate level of expertise to confirm that it is of an acceptable scientific standard.

# Reviewer report: Reviewer response for version 1

**DOI:** 10.5256/f1000research.156440.r297871

**Published:** 2024-07-24

**Authors:** Elizabeth Garcia

**Affiliations:** 1 Fundacion Santa Fe de Bogota, Bogotá, Bogota, Colombia

The present study offers key insights into the potential immunological cross-reactivity between human and fungal aquaporins.

- Major highlights include the discovery of substantial sequence and structural homology, which indicates possible sites for cross-reactivity, and the epitope analysis that identified overlapping regions among proteins, suggesting mechanisms for immune response triggers. The allergenic potential of aquaporins from various sources extends the study's implications, indicating similar underlying mechanisms in various allergic conditions, specially atopic dermatitis. The innovative integration of bioinformatics tools provides a comprehensive view of molecular mimicry, setting a precedent for future studies. Clinically, identifying specific epitopes that contribute to cross-reactivity opens avenues for targeted therapies and diagnostic tools, enhancing allergy management and prevention.
- Minor highlights include the rigorous methodology and the clear direction for future research, emphasizing experimental validation to confirm findings. This study, with its broad potential impact, notably contributes to understanding molecular mimicry in allergic diseases and sets the stage for future investigations in the field.

Is the work clearly and accurately presented and does it cite the current literature?

Yes

If applicable, is the statistical analysis and its interpretation appropriate?

Yes

Is the study design appropriate and is the work technically sound?

Yes

Are the conclusions drawn adequately supported by the results?

No

Are sufficient details of methods and analysis provided to allow replication by others?

Yes

Reviewer Expertise:

As an allergist and immunologist I have contributed to studies such as: “Potential contribution of Helicobacter pylori proteins in the pathogenesis of type 1 gastric neuroendocrine tumor and urticaria. In silico approach. PLOS ONE | https://doi.org/10.1371/journal.pone.0281485. April 25, 2023

I confirm that I have read this submission and believe that I have an appropriate level of expertise to confirm that it is of an acceptable scientific standard.

# Reviewer report: Reviewer response for version 1

**DOI:** 10.5256/f1000research.156440.r301819

**Published:** 2024-07-13

**Authors:** Celso Eduardo Olivier

**Affiliations:** 1 American Institute Alergoimuno, Americana, Brazil

It is a well-executed in-silico analysis comparing the amino acid sequences of human aquaporin 3  and *Aspergillus fumigatus* aquaporin with 25

 aquaporins from various allergenic sources researched from the UniProt and NCBI databases. However, in "background," "introduction," and "discussion," the authors assume that the IgE antibody exclusively mediates allergic diseases and hypersensitivities mechanisms. Recently, at least seven types (with several subtypes) of hypersensitivity reactions were postulated. I suggest the authors peruse and pay attention to the following references in order to refer appropriately to the subject: [ref 1, 2].

Is the work clearly and accurately presented and does it cite the current literature?

Partly

If applicable, is the statistical analysis and its interpretation appropriate?

Not applicable

Are all the source data underlying the results available to ensure full reproducibility?

Yes

Is the study design appropriate and is the work technically sound?

Yes

Are the conclusions drawn adequately supported by the results?

Partly

Are sufficient details of methods and analysis provided to allow replication by others?

Yes

Reviewer Expertise:

Allergy and Immunology; non-IgE-mediated hypersensitivity

I confirm that I have read this submission and believe that I have an appropriate level of expertise to confirm that it is of an acceptable scientific standard, however I have significant reservations, as outlined above.

## References

[1] : Nomenclature of allergic diseases and hypersensitivity reactions: Adapted to modern needs: An EAACI position paper. *Allergy* .2023;78(11) : 10.1111/all.15889 2851-2874 10.1111/all.15889 37814905

[2] Contribution of the Leukocyte Adherence Inhibition Test in Diagnosing Non–IgE-Mediated Immunoreactivity against Aspergillus fumigatus/authors.
